# Comprehensive review of the evidence regarding the effectiveness of community–based primary health care in improving maternal, neonatal and child health: 7. shared characteristics of projects with evidence of long–term mortality impact

**DOI:** 10.7189/jogh.07.010907

**Published:** 2017-06

**Authors:** Henry B Perry, Bahie M Rassekh, Sundeep Gupta, Paul A Freeman

**Affiliations:** 1 Department of International Health, Johns Hopkins Bloomberg School of Public Health, Baltimore, Maryland, USA; 2 The World Bank, Washington, District of Columbia, USA; 3 Medical Epidemiologist, Lusaka, Zambia; 4 Independent consultant, Seattle, Washington, USA; 5 Department of Global Health, University of Washington, Seattle, Washington, USA

## Abstract

**Background:**

There is limited evidence about the long–term effectiveness of integrated community–based primary health care (CBPHC) in improving maternal, neonatal and child health. However, the interventions implemented and the approaches used by projects with such evidence can provide guidance for ending preventable child and maternal deaths by the year 2030.

**Methods:**

A database of 700 assessments of the effectiveness of CBPHC in improving maternal, neonatal and child health has been assembled, as described elsewhere in this series. A search was undertaken of these assessments of research studies, field project and programs (hereafter referred to as projects) with more than a single intervention that had evidence of mortality impact for a period of at least 10 years. Four projects qualified for this analysis: the Matlab Maternal Child Health and Family Planning (MCH–FP) P in Bangladesh; the Hôpital Albert Schweitzer in Deschapelles, Haiti; the Comprehensive Rural Health Project (CRHP) in Jamkhed, India; and the Society for Education, Action and Research in Community Health (SEARCH) in Gadchiroli, India.

**Results:**

These four projects have all been operating for more than 30 years, and they all have demonstrated reductions in infant mortality, 1– to 4–year mortality, or under–5 mortality for at least 10 years. They share a number of characteristics. Among the most notable of these are: they provide comprehensive maternal, child health and family planning services, they have strong community–based programs that utilize community health workers who maintain regular contact with all households, they have develop strong collaborations with the communities they serve, and they all have strong referral capabilities and provide first–level hospital care.

**Conclusions:**

The shared features of these projects provide guidance for how health systems around the world might improve their effectiveness in improving maternal, neonatal and child health. Strengthening these features will contribute to achieving the goal of ending preventable child and maternal deaths by the year 2030.

Sustainability of effectiveness in improving maternal, neonatal and child health (MNCH) is an ideal that all MNCH programs seek. However, specially funded projects that undergo evaluation usually have a relatively short duration of five years or less. National demographic and health surveys may show long–term national improvements in child health, but determining the programmatic factors responsible for those improvements is difficult. As we have seen in this series of articles, the evidence regarding the effectiveness of community–based primary health care (CBPHC) in improving MNCH is based primarily on short–term assessments of a smaller group of selected interventions. Although two–thirds (66.7%) of the 152 maternal health assessments in our review were of projects with more than five interventions, only 15.8% of the projects were assessed for five or more years. Three–fourths of the 548 assessments of neonatal/child health projects included in our review assessed four or fewer interventions that were implemented over a period of less than five years.

However, one important question this review can address is: *What packages of community–based primary health care activities have produced evidence of long–term impact on MNCH?* A related question is: *Are there any common implementation strategies that these programs have in common that might help to explain their long–term effectiveness?* The answers to such questions can be helpful in considering how CBPHC can most effectively improve the health of mothers, neonates and children at scale over the longer term in high–mortality, resource–constrained settings.

The purpose of the current paper is to review the database assembled for the current journal supplement, of which this article is a part, and to describe the features of projects with more than one intervention that have evidence of long–term impact on maternal, neonatal or child health.

## METHODS

**Figure 1:**
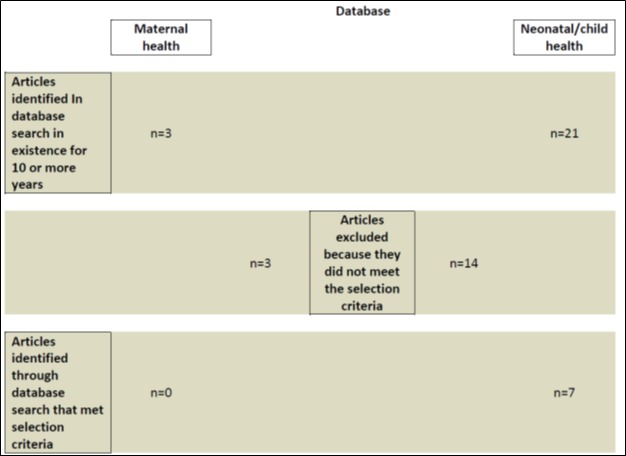
Selection of projects with long-term evidence of impact on maternal or neonatal/child health through integrated community-based primary health care (CBPHC).

The database of assessments of the effectiveness of CBPHC in improving MNCH has been described elsewhere in this series [1]. In short, it consists of data extracted from 700 documents describing the effectiveness of one or more interventions that have been implemented in the community outside of a health facility. Each assessment consisted of measurements of changes in maternal, neonatal and child health in terms of changes in population coverage of one or more evidence–based child survival interventions, in nutritional status, in serious morbidity, or in mortality. We queried this database for programs/projects/studies (hereafter referred to as projects) that had a duration of 10 years or more. Three assessments in the maternal health database were identified, and none of these met the criteria for this analysis. Twenty–one assessments in the neonatal/child health database were identified. Of these, 14 did not meet the criteria for this study for the reasons shown in **Figure 1**.

As shown in **Table 1**, 17 assessments of projects having a duration of 10 years or more were excluded from this analysis primarily because no measure of changes in mortality were available or the project implemented only one intervention.

**Table 1:** Reasons for exclusion of assessments of projects of 10 or more years’ duration

Reason for exclusion	Projects of 10–year duration or more excluded from analysis	
**Maternal database**	**Neonatal/child health database**
No measure of mortality included	1	5
No baseline measure of mortality	2	2
Mortality impact data covered less than 10 years of programming		2
Only 1 intervention implemented		3*
No evidence or mortality impact		1
No comparison area		1
Total	3	14

*Vitamin A in one assessment, malaria control in one assessment, and conditional cash transfers in another assessment.

The remaining seven assessments [2–8] that qualified for the analysis concerned four projects:
  - The icddrb MCH–FP project in Matlab, Bangladesh;
  - The Hôpital Albert Schweitzer in Deschapelles, Haiti (which operates a CBPHC program);
  - The Jamkhed Comprehensive Health Project in Jamkhed, India; and,
  - SEARCH (Society for Education, Action and Research in Community Health) in Gadchiroli, India.

Additional literature on these projects was reviewed, and additional findings were incorporated based on personal experience and field visits of one of the authors (HP) to these projects along with personal communications with persons engaged in these projects. By coincidence, Dr Perry worked in Bangladesh from 1995–1999 and visited the Matlab field site on a number of occasions. He served as Director General/CEO of the Hôpital Albert Schweitzer in Deschapelles, Haiti from 1999–2003. He has visited the CRHP project on four separate occasions (1998, 2004, 2006 and 2009) and the SEARCH project on two occasions (2004 and 2006).

## RESULTS

The four projects identified from our database that had evidence of under–5 mortality impact for 10 years have each been functioning for 30 years or longer and are still functioning. These projects also had considerable evidence of improvements in coverage of key evidence–based interventions. These four projects are:

- The icddrb MCH–FP project in Matlab, Bangladesh (a maternal/child health and family planning research field site for icddrb, formerly known as the International Centre for Diarrhoeal Disease Research, Bangladesh);
- The Hôpital Albert Schweitzer in Deschapelles, Haiti;
- The Jamkhed Comprehensive Health Project in Jamkhed, India; and,
- SEARCH (Society for Education, Action and Research in Community Health) in Gadchiroli, India.

Here we describe below the main features of these projects, recognizing that over such a long period of time these features have not remained static. Nonetheless, the descriptions are appropriate for the time in which the mortality impacts were achieved even though they may not entirely accurately describe current activities.

### The Icddrb MCH–FP project in Matlab (Bangladesh)

#### Project description

The Cholera Research Laboratory (CRL) was established in 1960 in Dhaka, Bangladesh to develop methods for preventing and treating cholera. In 1963, the CRL established a field site in a rural riverine area three hours southeast of Dhaka in a cholera–endemic area to test new approaches for controlling the disease, including the testing of the effectiveness of new cholera vaccines. In 1966, a Demographic Surveillance System (DSS) was established at Matlab with the initial goal of assessing the impact of new vaccines on morbidity and mortality. The DSS has become the oldest demographic surveillance system in the world, and Matlab is the site of hundreds of field research projects regarding health, nutrition, population and socio–economic development. The CRL expanded its work to maternal/child health and family planning in 1977, and in 1978 the Government of Bangladesh established the International Centre for Diarrheal Disease Research, Bangladesh (now icddrb), which took over responsibility for the Matlab DSS and field activities [9–11].

The field site is divided into two parts. The first is an Intervention Area, where intensified community–based health and family planning activities operated by icddrb began in 1977. This is the Maternal–Child Health and Family Planning (MCH–FP) project. The second is a Comparison Area, where only government health services are provided. Each of these two areas has a population of approximately 112 000 persons.

Eighty paid community health workers (CHWs) in the Intervention Area visit each home on a regular basis. (The frequency of visits has declined gradually from every two weeks in 1977 to every two months at present.) Each CHW is responsible for approximately 200 households and typically visits 20 homes per day. At the time of a home visit, the CHWs immunize women and children, provide antenatal and postnatal care, and treat childhood pneumonia according to WHO guidelines. They provide nutrition education and treat diarrheal disease. They also leave packs of oral rehydration salts (ORS) with a “depot holder,” who is a mother in the neighborhood with additional training in the treatment of childhood diarrhea. Finally, the CHWs promote family planning, distribute birth control pills and condoms, administer injectable contraceptives and track pregnancies.

The CHWs working in the icddrb MCH–FP project are well–trained and well–supported, and they can refer patients to a nearby sub–center staffed by a full–time paramedic who provides routine maternal and child health care as well as reproductive health care. A hospital operated by the project is readily available for referrals. This referral system and readily available hospital care was a key element of the initial CRL activity since the survival of patients with cholera depended on prompt identification and transport, usually by boat in this riverine environment, to the hospital in Matlab operated by the CRL. The project earned a high level of trust with the population because of the high quality of health care it has provided over four decades. Maintaining good relations with the community is a priority for the Matlab MCH–FP project, and project managers promptly address any issues raised by the community about the quality of services or the nature of the field research activities. The total annual cost per capita for the community–based portion of the health project (excluding research–related expenses) is about US$ 5 [12].

Four sub–centers are located in the Intervention Area (one for about 28 000 people), and 20 CHWs are assigned to each sub–center, where a full–time paramedic works. CHWs meet at the sub–center every two weeks for supervision, continuing education, and replenishment of supplies. Basic comprehensive primary health care is provided by the paramedics, including insertion of IUDs, menstrual regulation (suction curettage of the uterus for women with delayed menstrual periods who do not want to become pregnant), and treatment of sexually transmitted diseases and reproductive tract infections. Icddrb also operates a 50–bed inpatient facility that serves the Intervention Area. A government district hospital serving a larger geographic area is also in Matlab. Major surgical procedures are not available at the icddrb Matlab facility, but emergency obstetrical care, including caesarian section, is provided in collaboration with the government district hospital in Matlab [12].

Key components for success at Matlab include:

- Sound organizational structure from the outset;
- Readily available transport throughout the project area, mostly by speedboat, which has facilitated patient referral to the Matlab Hospital;
- A strong system of accompaniment and support for all levels of workers;
- A well–developed record–keeping system; and
- Continuously available supplies.

The book *Matlab: Women, Children and Health* provides a full discussion of the history of Matlab, its operations and research findings through the early 1990s [10].

#### Long–term outcomes

In 1984, the contraceptive prevalence rate (CPR) in the Intervention Area was 46% compared while it was 16% in the Comparison Area and 19% nationwide. In 2005, the CPR in the Intervention Area was 71%, 47% in the Comparison Area, and 58% nationwide. In 1987, the coverage rate for the standard series of childhood immunizations was 69% in the Intervention Area compared to a national rate of approximately 20% nationwide [12]. In 2005, the childhood immunization coverage rate in the Intervention Area was 97% compared to 85% in the Comparison Area.

Between 1988 and 1993, the mortality rate from pneumonia in children younger than 2 years of age was 54% lower in the Intervention Area than in the Comparison Area [13]. There was a reduction by around 75% in the annual number of childhood deaths over a 25–year period in Matlab, and over a 40-year–period, life expectancy increased from 50 to around 65 years [10].

The infant and 1– to 4–year mortality rates for the Intervention Area of MCH–FP project area were consistently lower than in the government services area (the Control Area) over a 15–year period between 1978 and 1994 [14,15]. In 1985, the under–five mortality rate (U5MR) per 1000 live births was approximately 200 in the Comparison Area and 150 in the Intervention Area (25% less). In 1995, the rates were approximately 120 and 75 respectively (38% less in the Intervention Area). In 2005, the under–five mortality rate was 46.6 in the intervention area and 62.4 in the Comparison Area (25% less) [14,15]. Over the period from 1982 to 2005, the maternal mortality rate (that is, the number of maternal deaths per 100 000 women of reproductive age) was 37% lower in the Intervention Area than in the Comparison Area, mainly as a result of a lower pregnancy rate and lower case–fatality rates for induced abortion, miscarriages and stillbirths [16].

The total fertility rate (TFR) over time has been the following: in 1985, the TFRs were 4.5 in the Intervention Area and 6.0 in the Comparison Area; in 1995, they were 3.0 and 3.6 respectively; by 2005, the rates were essentially the same at 2.7 and 2.8, respectively [14,15].

The progress in the Control Area can be attributed in part to the national application of the Matlab family planning model of home visits by paid workers to promote the use of family planning and the distribution of birth control pills and condoms. By the mid–1980s, Bangladesh essentially had a national CHW program. Progress in increasing the use of facilities for giving birth was slower in the Comparison Area. In 2004, only 12% of the births in the Comparison Area were taking place in a facility while in the Intervention Area the corresponding figure was 50% [14,15].

#### Lessons learned

Two lessons learned at Matlab and reported in 1994 bear emphasis here:

- “Family planning field workers are more likely to gain the confidence of the community if they respond to other health problems, particularly those of women and children…. [T]he benefits of integrating quality health services into a family planning programme justify the heavy inputs” [17].
- “The successful operation of such a large and multifaceted project as Matlab requires a professional level of organization in the hands of a competent manager. This applies for staff management, logistics and supplies, and relations with the community” [17].

One of the striking findings from the Matlab example is how quickly child mortality in the Comparison Area declined and how the difference between the Intervention and Comparison Areas gradually narrowed later. The differences in mortality rates for infants and children between the Intervention and Comparison Areas have narrowed over time. This can partly be explained by the fact that the MCH–FP Project at Matlab served as a model of CBPHC for the country, and Bangladesh has done a masterful job of extending home–based services – both MCH and FP services – throughout the country. Bangladesh is one of only 19 out of 68 high–mortality countries that reached the Millennium Development Goal for children by 2015 [18], and its national achievements in expanding coverage of community–based services has been widely documented and applauded [19]. After the interventions of the Matlab MCH–FP Project were proven to be effective in the 1970s, there was an explicit effort in the 1980s to introduce this same strategy nationally, with obvious benefits.

### Hôpital Albert Schweitzer (Deschapelles, Haiti)

#### Project description

L’Hôpital Albert Schweitzer (HAS) began operations in 1956 after a wealthy American couple, William Larimer and Gwen Grant Mellon, were inspired by the example of the great medical missionary Albert Schweitzer who, for more than a half–century, provided medical care in Gabon, an underserved country of West Africa. The Mellons constructed one of Haiti’s first modern hospitals in the Artibonite Valley, three hours northwest of capital, Port–au–Prince [20].

For the first decade of its existence, HAS provided only hospital care and services at an outpatient clinic based at the 190–bed hospital. In its second decade (in 1967), it established a project of community–based primary health care based on community health workers (*agents de santé*) and mobile health teams without any peripheral primary health care facilities. Over time, seven health posts and two health centers opened. The hospital always served as the Ministry of Health’s district hospital for the health district in which it is located, with 258 000 people in its catchment area during most of the period covered by the impact assessment. The population served by HAS’s primary health care project fluctuated over the years, from 18 820 in 1958 to 180 000 in 1996 and to 350 000 in 2016 [3,20].

In the 1960s, HAS also established community development activities, including projects for improving water and sanitation at the village and household levels, promoting vegetable gardens and reforestation, providing opportunities for micro–credit and income–generation for women, literacy training, support for primary education, and promotion of animal husbandry and improved agricultural production. HAS thus became a comprehensive integrated health and development system with strong CBPHC services together with facility–based primary health care, hospital referral care and community development activities [3].

The CBPHC services at HAS have relied on paid Health Agents (*Agents de Sante*) who regularly visit every home to provide basic health education, register vital events, and mobilize mothers and children to attend Rally Posts where essential services are provided, including immunizations, growth monitoring/promotion, and referral care at the hospital. Mobile clinics reach all isolated areas intermittently. These are staffed by an auxiliary nurse who, every 1–2 months, visits isolated communities on foot (since there are few roads in the mountains) to provide basic curative and preventive care (including family planning) and to refer patients when needed.

In the late 1990s, 1500 volunteer community health workers (*Animatrices*), one for every 15 households, were recruited to provide peer–to–peer health education to other women, to assist with the Rally Posts and Mobile Clinics, to promote community involvement, and to assist with referral of patients to higher levels of care [3]. In addition, eight Monitors (*Monitrices*) provide liaison with and training of lay midwives, along with supervising and training the *Animatrices.* The role of *Monitrices* at HAS initially involved supervising the community–based nutritional rehabilitation project, known as the Hearth Project, which originated at HAS and has been implemented in numerous other countries. Finally, the CBPHC services at HAS include 16 community–based tuberculosis workers (seven *Accompagnateurs* and nine *Agents*) who obtain sputum specimens from symptomatic patients and provide directly observed therapy for patients in their home. Community–based provision of anti–retroviral medication for patients with HIV/AIDS is now provided as well.

Steady financial support from external donors has been available to HAS since its inception, and this has helped HAS to provide high–quality professional leadership and management. It has been able to ensure logistical support for its field projects and to provide needed supplies and drugs. The quality of its clinical services has earned the trust and support of the population over a long period of time. The hospital is widely regarded as one of the best district hospitals in a rural area of a developing country, and patients from throughout Haiti have come there for treatment.

#### Long–term outcomes

In 2000, population coverage rates of key child survival interventions in the HAS primary health care service area were approximately twice those for the same interventions nationwide in rural Haiti. Additionally, the U5MR in the HAS service area was less than half of that for Haiti overall (62.3 vs 149.4) [3]. Likewise at that time, the CPR in the HAS project area was nearly double that in other areas (27.5% vs 15.4%) [3]. Great efforts have been made to ensure access to basic services in the most isolated parts of the HAS project area, some of which require eight–hours by foot to reach.

As a result of the collection of vital events data at the time of initiation of HAS’s community health project in 1967 [21] and the intermittent collection of retrospective birth histories since, it has been possible to monitor the U5MR for the primary health care project area served by HAS and to compare these to data for Haiti as a whole. The HAS project area is similar in socio–economic indicators to rural Haiti as a whole [4].

The U5MR in the HAS primary health care service area remained much lower than in Haiti nationally over a three–decade period from 1970 to 1999 [4]. The rapid decline in under–five mortality to one–quarter of the national level between 1958 and 1973 was due in large part to the elimination of neonatal tetanus through immunization of all women of reproductive age [22,23]. Between 1970 and 1999, the U5MR remained less than half that of the U5MR for Haiti [4].

The per capita annual cost for the entire project as it existed in 1999 would be US$ 24.77 in 2016 dollars. Because of resource constraints, the projects at HAS have undergone significant cutbacks over the past decade. The cost per under–five death averted in current dollars was US$ 3233; the cost per year of live saved was US$ 47; and the cost per DALY saved was US$ 90 [24].

#### Lessons learned

There does not appear to be any single intervention or even a small set of interventions responsible for the sustained mortality reduction. Rather, the entire system of health and development – community–based services, primary health care services at health posts and health centers, hospital services, community development projects, as well as the interactions between these elements – most likely contributed to this mortality impact. The close integration of the CBPHC activities with the primary health care facilities and the hospital are key elements of system effectiveness.

The rapid decline of mortality for Haiti as a whole during the period from 1970–1999 is worth noting, particularly in light of the country’s political instability, its deteriorating economic situation, and the epidemic of HIV/AIDS throughout the country, which began in the early 1980s. In fact, in spite of the devastating earthquake in the capital in 2010 and more recent cholera outbreaks, Haiti is one of only 34 of the 74 so–called Countdown Countries (with 97% of world’s maternal and child deaths) to have achieved the Millennium Development Goals in 2015 for reduction in child and maternal mortality [25]. The model of CBPHC developed at HAS is now utilized by virtually all other NGOs working in community health in the country, and these NGOs provide community–based child survival services to two–thirds of the population of Haiti. The nationwide contribution of CBPHC to the gains achieved in child survival in Haiti have been possible in part because of the early experience at HAS and its position as a role model for the rest of the country.

### India: the Jamkhed Comprehensive Rural Health Project

#### Project description

The Jamkhed Comprehensive Rural Health Project (CRHP) in Ahmednagar District of Maharashtra, India, has been in operation for almost five decades [5,26]. It developed a comprehensive approach to community–based health programming in conjunction with first–level hospital referral services. Its principles of equity, integration and empowerment have been guiding principles throughout this prolonged period.

When CRHP began in 1970, the people of the Jamkhed area were living in near–famine conditions from drought and lack of access to water. The prevalence of childhood malnutrition was 40%, and coverage rates of childhood immunizations, family planning, prenatal care, and birth attendance by a trained provider were all less than 1%; and the infant mortality rate was 176 per 1000 live births. The caste system was ingrained, and harmful traditional practices, especially for women, were common. In addition, women had no personal rights. Furthermore, they were often treated inhumanely. One–third of the population was migrating to sugar cane plantations outside of the district to work in temporary jobs because of the scarcity of food and the lack of work in the Jamkhed area.

Rajanikant and Mabelle Arole started working in Jamkhed in 1970 as a husband–wife physician team treating patients who came to them with medical problems. They quickly realized that over three–quarters of health problems could be addressed at the community level, mainly by the villagers themselves, if they had a modest amount of additional knowledge and skills. The main purpose of their work soon became to facilitate a process whereby communities could improve their health through their active participation by learning about and addressing their problems based on their own priorities.

Some of the initial activities carried out were: health promotion through health education, immunization, prenatal care, complementary infant feeding, ensuring safe delivery, family planning, and a health center for curative care. Their work gradually expanded to train illiterate CHWs, address the determinants of ill–health through improving access to water and food, nutrition education and kitchen gardens, women’s and community empowerment, micro–credit, education, improved agriculture, and prioritizing the needs of the poorest and most disenfranchised members of communities. From the beginning, CRHP worked only with communities that requested assistance and committed themselves to participation. Gradually, all villages in the area sought to be involved as they saw the benefits to other communities from participation.

CRHP always insists on major investments of time and energy from community members as a condition of CRHP’s entering into partnership with a community, so the process that emerged ensures future sustainability. The project established groups of volunteers within the community, including village health workers (VHWs), farmers’ clubs and women’s groups (*mahila mandals*), and, more recently, girls’ and adolescent boys’ groups.

The key change agent in the community became the VHW, who is selected by the community. She is eager to assist her village, especially the poorest and most marginalized members such as *Dalits* (untouchables) and those with stigmatized conditions (such as leprosy). She receives training in health, community development, communication, organization and personal development. Her primary role is to share her knowledge with everyone in the community, to organize community groups, and to facilitate the community’s assessment of problems and resources, analysis of causes and determinants, and appropriate actions, especially with the poor and marginalized that might be undertaken with the assistance of CRHP. Initially, many of these VHWs were illiterate women from the untouchable (*dalit*) caste who had recovered from an illness (such as tuberculosis) as a result of care provided by CRHP.

Although the VHWs do not work for pay, with project assistance they obtain access to income–generating activities. They serve as a link between the community and the project’s mobile team, which visits each village once a month or more often if needed. The mobile team consists of a nurse, an agricultural specialist and a social worker, though they all become multipurpose workers through working together, learning from each other, and additional training.

The VHWs come to the project center in the small town of Jamkhed once a week. There they meet with the other VHWs to discuss problems encountered in their work and to obtain further training from each other and from staff. They spend the night there and provide social support for each other. Many of the VHWs have been working for more than 30 years. Dropouts are rare, mainly because of old age and death.

For many years, CRHP operated a 30–bed hospital that served as a referral source for patients from the project area and beyond. A larger 50–bed hospital has recently been completed. Emergency cesarean section and other emergency surgical procedures are performed there. At the beginning of the project, the hospital in Jamkhed was filled with children who had life–threatening infections and malnutrition. Such patients are rarely seen there now.

CRHP gradually expanded to reach 300 villages with a population of 500 000 people. Most of these villages are now independent, thanks to the sustainable development process that CRHP has nurtured over five decades, so CRHP now focuses on the villages that need them most.

Because of the great interest of people throughout India and the world to learn about the CRHP experience, the Jamkhed International Institute for Training and Research in Community Health and Development was established in 1992. More than 30 000 people from throughout India and more than 3000 people from over 100 countries have come there to learn from the VHWs, other villagers and CRHP staff and to visit villages to see the impact firsthand.

Each village maintains a record of all births and deaths that take place among its members, as well as records of the number of eligible couples who are using family planning, the number of children completely immunized, and the number of children with malnutrition. Also included is information about socioeconomic conditions, agricultural and environmental issues, and various priority diseases. This information is written on a board that is displayed in a public space in the village and services as a focal point for discussions about priorities for the community to address. Participatory Rural Appraisal (PRA) techniques are commonly used for assessments and analysis as well as for discussions on what to do. All segments of the community participate.

#### Long–term outcomes

By 1993, the percentage of pregnant women with antenatal care and a safe delivery reached 82% and, in 2011, it reached 99%. The percentage of couples utilizing family planning reached 68% in 2004. In 2004, 87% of children were fully immunized and only 5% were undernourished according to anthropometric measurements. This low prevalence of undernutrition has been maintained ever since. Leprosy, which was common at the start of the project, has virtually disappeared, and the incidence of tuberculosis has declined from 1800 to 200 cases per 100 000 persons [26].

The IMR at CRHP Jamkhed declined from 176 deaths per 1000 live births in 1971 to 19 in 1993 [5] to 8 in 2011, according to data collected at CRHP by CHWs [26]. In 1971, the IMR at CRHP Jamkhed was 60% greater than for the rural area of the state of Maharashtra (176 vs 110), but since 1980 the IMR at CRHP Jamkhed has been half that for rural Maharashtra [27]. A large–scale external and independent evaluation of the mortality impact of CRHP based on a comparison of findings from birth histories in project villages with those in a surrounding control area was carried out in 2007–8. This evaluation demonstrated a 30% reduction in the risk of death among children 1–59 months of age in CRHP project villages compared to control villages [28].

Although baseline levels of maternal and perinatal mortality were not measured in the 1970s when CRHP began, these rates were measured following a careful review of all births and deaths in 25 villages around Jamkhed between 1996 and 1999. A maternal mortality ratio of 70.0 per 100 000 live births and a perinatal mortality rate of 36.0 per 1000 live births and stillbirths were measured at CRHP [29]. These rates were 27.8% and 20.3% lower respectively than the maternal and perinatal mortality for Pune district in Maharashtra State in India, were the CRHP is located [29].

These significant results were accomplished because of the communities’ participation and empowerment together with their understanding of health promotion and disease prevention. For example, family members know the importance of healthy nutritional practices, prenatal care, how to provide early home care for common problems (such as homemade oral rehydration solution for diarrhea, steam inhalation for respiratory problems, sponging with cool water for fever, and sunlight for neonatal jaundice). VHWs ensure exclusive breastfeeding for infants during their first 6 months of life, proper burping after feeding, and nutritional weaning foods. The men and women’s groups weigh the children for growth monitoring. Immunizations were also gradually accepted by the communities as the program developed. The government now provides these services with the support and cooperation of the VHWs and community groups.

In the early years the communities organized feeding programs for groups of children, with everyone contributing something (eg, firewood, water, salt, grains, or pulses), and the Farmers’ Clubs dedicated some of their land for growing food for the program. They established watershed development projects to increase the available of groundwater for home and agricultural use. Most homes now have kitchen gardens for additional nutritious fruits and vegetables.

#### Lessons learned

To be sure, the impact of CRHP is demonstrated through changes in health statistics, which show positive results achieved over more than four decades. Behind these statistics are self–confident men and women, once outside the mainstream of society, taking leadership positions in their villages, affirming that they have God–given dignity, worth and capacity. Thus, it is not only the quantitative changes that are important. Even more important is the transformation of persons and communities in a qualitative way, which have made these health improvements possible.

Community empowerment increases self–reliance, self–esteem, self–confidence – and it reduces dependency on outside agencies. In order for the development process to be sustainable by the people, the community must have good leadership and the capacity to address its own issues. In the Jamkhed process, the community learns to work together and solve problems together. If the community needs more knowledge, skills or resources, CRHP helps them.

The Jamkhed process of sustained health improvement through CBPHC involves:

- Expanding knowledge and skills through building the capabilities of individuals and communities, based on where they are and what they have.
- Developing a caring and sharing community that promotes reconciliation and peace (*shalom*) by engaging the whole community, including the poorest and most marginalized members and integrating them as active members of the community to solve the problems that concern them most through assessment, analysis, and action.
- Promoting volunteerism by building a community of motivated and caring individuals committed to engaging in these activities.
- Focusing on low–cost activities including home remedies and herbal medicines as well as health promotion, prevention, early detection, treatment, and rehabilitation in the community.
- Utilizing appropriate technology and local resources that are accessible in the context of the community’s knowledge, skills and interests.
- Engaging in multi–sectoral development, including education, sanitation, and income generation, as well as building social capital and helping people to recognize the harm that some traditional practices are causing for the purpose of improving the overall well–being of the community, recognizing that conditions outside of the health sector have more impact on health than curative care alone.
- Recruiting, training and supporting women VHWs, who are so motivated that after decades of service they are still active leaders, still learning and sharing with their communities and others.

This transformative process is spread to other communities by the villagers who have experienced it, making it a people’s movement. This is not an innovation in technology but rather an innovation from within each community that brings about social change and thereby uplifts everyone from poverty and disease. Lives are transformed by embracing the dignity and worth of everyone and giving an opportunity to all to contribute.

CRHP is one of the world’s leading examples of improving MNCH through community empowerment, women’s empowerment and community participation. In spite of not being well–known in academic and research circles, it is well known in the broader global health community through the visits of thousands of people from throughout India and around the world as well as through the 1994 publication of the acclaimed book by the Aroles, simply entitled *Jamkhed: A Comprehensive Rural Health Project* [5]. This book is one of the best long–term sellers among global health books and has been translated into a number of different languages.

Of historical importance is the fact that the CRHP served as one of the inspirations for the 1978 International Conference on Primary Health Care at Alma–Ata. CRHP was one of the projects featured in the influential monograph published by the World Health Organization several years prior to the Conference [30,31]. In contrast to the limited information in most peer–reviewed scientific articles regarding the context within which CBPHC operates and how it is actually implemented in the community, there is extensive information about these benchmarks in the acclaimed book by the Aroles [5].

### SEARCH (Society for Education, Action and Research in Community Health) in Gadchiroli, India

#### Project description

Since 1986, the Society for Education, Action, and Research in Community Health (SEARCH) has provided community–based health care services and hospital care in a rural area of the state of Maharashtra, India, known as Gadchiroli [32,33]. The Gadchiroli District is the least developed in the state. The district is largely forested, and half of the inhabitants are indigenous tribal people who live in the forest. The other half is composed predominantly of Hindu subsistence farmers. Gadchiroli is 175 km south of Nagpur in the most western part of Maharashtra.

The founders, Dr Abhay Bang and Dr Rani Bang, were inspired by the life of Mahatma Gandhi and established their work in the context of Gandhian social philosophy. They developed a collaborative partnership with the communities of Gadchiroli for basic health care, education and training in health, and for research to inform health policies [34]. Like the Aroles, who founded the Jamkhed CRHP Project, the Bangs obtained important insights for their work from the Narangwal Project, a model community health project established in collaboration with the Johns Hopkins University in the 1970s [35,36].

The Bangs established three goals for their organization: (1) provide health care to the local population, (2) provide training and education in health, and (3) conduct research to shape health policies. The vision of SEARCH is the realization of *Aarogya–Swaraj* (translated as “the people's health in people's hands”) by empowering individuals and communities to take charge of their own health, thereby helping them achieve freedom from disease as well as from dependence. The mission of SEARCH is expressed in its name, “Society for Education, Action and Research in Community Health.” The mission is “to work with marginalized communities to identify their health needs, develop community–empowering models of health care to meet these health needs, to test these models by way of research studies, and then to make this knowledge available to others by way of training and publications” [32]. Thus, community–based primary health care, community participatory research and training of village people are core activities at SEARCH.

Over the past 30 years, Drs. Abhay and Rani Bang and their dedicated staff developed a community health project that provides community–based primary health care for a population of 80 000 people. One–half of this area is used as a field site for implementing new interventions while the other half serves as a control area in the sense that the new intervention is not being implemented there during the study period. SEARCH also operates a 20–bed hospital and outpatient facility to serve tribal people from the area.

SEARCH pioneered the development of a community–based reproductive health care project and related research. It also developed a pioneering community collaboration to address alcohol and drug addiction, which was initiated in response to requests from the community [37]. Basic surgical services are provided at the hospital, including cesarean sections and surgical care for a common cause of long–term disability – massive hydrocele caused by lymphatic filariasis. Patients requiring higher levels of care are transported to a government hospital in the city of Gadchiroli, which is about 30 minutes away. The staff at SEARCH consists of 30 members, including physicians, paramedics, project supervisors and managers, and research staff.

SEARCH established a partnership with communities over the past two decades by listening to members, responding to their expressed concerns and priorities, and involving them in the planning, implementation and evaluation of its projects. The community has taken co–ownership of the project.

SEARCH does not duplicate the government health system. Instead, it has developed a community–based health provision system that utilizes the government health system for referrals. SEARCH employs, trains and supervises one female community health worker (CHW) for approximately every 1000 population. This CHW visits every home on a monthly basis, registers pregnancies, births and deaths since the previous visit, and provides health education and basic preventive and curative health care. By maintaining close contact with the households for which she is responsible, the CHW is able to provide childhood pneumonia treatment and home–based neonatal care along with other basic health care services for mothers and children. Between 1988 and 2005, SEARCH also provided strong training and support for the traditional village midwives – *dais*.

The Bang’s groundbreaking research on the effectiveness of community–case management of childhood pneumonia [38] and on the effectiveness of home–based neonatal care [2] has had a major impact on health care programs throughout the developing world. The project relies on trained traditional birth attendants and community health workers for diagnosis and treatment of common illnesses, diagnosis and treatment of childhood pneumonia, and provision of home–based neonatal care.

#### Long–term outcomes

The infant mortality rate in the Intervention Area declined by 74%, from 120 deaths per 1000 live births in 1988 to 31 in 2003 [2,39]. In the Comparison Area, over the period of time for which data have been reported (1994–2004), the IMR remained essentially unchanged [2,39]. This was the period during which the home–based neonatal care intervention was being implemented and evaluated.

#### Lessons learned

The pioneering findings of the community case management of pneumonia and of home–based neonatal care by SEARCH in Gadchiroli have stimulated much additional work by others around the world since the efficacy of these interventions were first reported by the Bangs in the 1990s [38,40]. The Bangs provided leadership for replication of the home–based neonatal care intervention by other NGOs in the state of Maharashtra, and they provided technical assistance for scaled–up versions of the SEARCH model for home–based neonatal care now being tested by the India Council of Medical Research at various sites around India.

Among other things, their work has demonstrated that properly trained, supervised and supported CHWs, even if they are illiterate, can provide high–quality technical interventions for mothers and children. The methods of selection, training and support of CHWs used by SEARCH merit closer analysis and widespread application.

## DISCUSSION

The Matlab MCH–FP project in rural Bangladesh, the HAS integrated project of health and development in rural Haiti, the Jamkhed CRHP in rural India, and the Gadchiroli SEARCH project in rural India are among the few examples that exist of projects with evidence of long-term reductions in maternal, neonatal or child mortality resulting from community–based interventions. Three of the four of these projects have been in operation for more than four decades, while the fourth (SEARCH) has been in operation for more than three decades.

### Common characteristics of projects with evidence of long–term impact on mortality

What is particularly striking is the similarity of many of the features of these projects. As **Table 2** demonstrates, all four of these projects are similar in the broad range of services they offer along the continuum of care for individuals at various points in the life cycle – from pregnancy and childbirth to the neonatal and child periods to adolescent and adulthood. They are also similar in the breadth of types of services – from preventive to curative to rehabilitative services. Finally, they are similar in the vertical integration of their services – from home–based and community–based services all the way to hospital referral services.

**Table 2:** Common characteristics of four projects with long–term evidence of impact on child mortality*

Characteristic	Hôpital Albert Schweitzer (Haiti)	Matlab MCH–FP project (Bangladesh)	CRHP–Jamkhed (India)	SEARCH–Gadchiroli (India)
**Basic project characteristics:**				
Year established	1956	1965	1970	1986
Population of catchment area	150 000	100 000	300 000	80 000†
Range of services provided:				
Is a comprehensive array of child health services provided? These include health and nutrition education, diagnosis and treatment of acute childhood illness, referral of seriously ill children to a higher level of care.	Yes	Yes	Yes	Yes
Is a comprehensive array of maternal, reproductive health, and family planning services provided? These include health and nutrition education, provision of antenatal care, management and/or referral of obstetrical complications, provision of postnatal care, and provision of a wide range of family planning methods	Yes	Yes	Yes	Yes
Are general curative services provided? These include treatment of common childhood illnesses and management (including referral when indicated) of serious childhood illnesses in the community; care for acute illnesses among patients of all ages in health centers, and referral of seriously ill patients to higher levels of care.	Yes	Yes	Yes	Yes
Are surgical and/or other hospital inpatient services provided?	Yes (operates its own first–level referral hospital with advanced surgical capabilities)	Yes (operates its own first–level referral hospital with no surgical capabilities)	Yes (operates its own first–level referral hospital with advanced surgical capabilities)	Yes (operates its own first–level referral hospital with some surgical capabilities, eg, cesarean section)
How strong is the referral system from the community to higher levels of care at fixed facilities, including hospitals? In all four projects, a first–level referral hospital is integrated into the project. However, all surgical cases at Matlab are referred to the government district hospital as are more complicated surgical cases at Jamkhed and SEARCH.	Very strong	Very strong	Very strong	Very strong
**Health project management and support:**				
Does the project have a strong system of management and supervision led by competent and dedicated professionals?	Yes	Yes	Yes	Yes
Does the project have a record of accomplishment in treating patients and clients with a high level of respect?	Yes	Yes	Yes	Yes
Does the project have a record of maintaining supplies and drugs?	Yes	Yes	Yes	Yes
**Nature of community partnerships/community involvement:**				
How strong is the partnership between the project and the community?	Fairly strong	Fairly strong	Very strong	Very strong
How strong is the level of trust of the community in the project?	Very strong	Very strong	Very strong	Very strong
**Role of community–based workers:**				
Are CHWs an integral part of the project?	Yes	Yes	Yes	Yes
Do CHWs receive financial support?	Yes	Yes	Yes‡	Yes
How strong is the training and support of CHWs?	Very strong	Very strong	Very strong	Very strong
Do CHWs have routine contact with all families through visitation of all homes?	Yes	Yes	Yes§	Yes
Do CHWs provide essential child health services in the home?	Yes	Yes	Yes	Yes

CHW – community health worker

*Some of this information is based on the authors’ field observations and discussions with project leaders and is not contained in written documents.

†The part of the SEARCH project area with documented declines in infant mortality has 40 000 people.

‡Although the CRHP CHWs do not receive a salary, they do receive special training and access to credit to enable them to become economically self–sufficient through their own income–generating activities. CRHP ensures that their CHWs have enough income to meet their needs.

Another characteristic these projects have in common is that they all have a strong community–oriented health system in which the community is a partner. Improving MNCH is one of many goals of the health system that these projects developed. However, they all also provide comprehensive primary health care services with a strong focus on maternal and reproductive health and family planning. They all provide hospital services and ensure that basic surgical care is available to the populations they serve. They all recognize the importance of a functioning referral system to ensure that patients can access higher levels of care when needed. Most importantly, all four of these integrated comprehensive projects have established strong CBPHC services that serve as a foundation upon which the other project activities rest. These CBPHC services all include strong collaborations and partnerships with communities.

All projects have strong professional leadership as well as dynamic management and supervisory systems. They ensure that essential supplies and drugs are available. They all have a record of treating patients with a high level of respect.

The projects have been developed and sustained with a high level of community engagement; the community has a high level of trust in the health services provided by the projects. The provision of a broad array of high–quality curative services by each of these projects over a long period of time has resulted in trust being developed with the communities.

A final important similarity is that all four projects created strong roles for community–level workers. The projects all realized their effectiveness would be compromised without building a central role for these workers, all of whom receive some type of financial assistance. These CHWs all receive high–quality training and supervisory support. They maintain routine contact with all families in service areas, and they provide essential health care in their homes and at readily accessible sites in the community and nearby.

These four projects have all influenced thinking and practice in CBPHC programming for MNCH around the world – through their research as well as through their influence on younger people who have had personal experiences in the field with these projects who later become global health leaders. And, of course, CRHP’s influence on the emergence of primary health care as defined at Alma–Ata as well as on the later emergence of national CHW programs in India is well–known [31,41].

The findings reported in this paper have focused on long–term improvements in neonatal and child health. But, it is important to point out that two of the four projects included here also have evidence of long–term reductions in maternal mortality: Matlab MCH–FP [10] and CRHP [29]. Exploring these findings in detail is beyond the scope of this article, but suffice it to say here that presence of strong CBPHC interventions for reproductive and maternal health (including family planning) linked to well–developed referral systems and readily available hospital care serve as the foundation for preventing maternal deaths.

Another interesting shared characteristic is that each of the four projects has a “culture” of science and evaluation, which led to the reporting of outcomes and the inclusion of these four projects in our review. These projects have been at the forefront of generation of knowledge about effective programming based on their field experiences.

A final shared feature of these four projects is their strong connection to the Narangwal Project and Dr Carl Taylor. We noted previously that both CRHP and SEARCH were directly influenced by the Narangwal Project, a pioneering field project in north India during the late 1960s and early 1970s that was one of the first to carefully evaluate the effectiveness of community–based primary health care [26,35]. The Aroles and the Bangs were master of public health students of Dr Carl Taylor’s at the Johns Hopkins University, where they learned about the Narangwal Project. The CBPHC work at icddrb was directly influence by the Narangwal Project as well because the director of fieldwork for that project, Dr Shusham Bhutyia, later initiated the training and support of CHWs for the Matlab MCH–FP project. The CBPHC work at HAS in Haiti was led by Drs. Warren and Gretchen Berggren, who were mentored in this by Dr John Wyon, a colleague of Dr Carl Taylor’s in north India and the field director for the Khanna Study [42], a community–based field research project that served as a predecessor of the Narangwal Project. Dr Carl Taylor served as a mentor to John Wyon during the development and implementation of the Khanna Study and they remained close colleagues subsequently.

However, in spite of these many shared characteristics, there are important differences to note as well. There are notable differences in the four projects in terms of the degree to which they have engaged in research and reported their results in peer–reviewed journals. The Matlab MCH–FP project is one of the world’s foremost field research sites. SEARCH has been the site of some of the most influential research in global health related to community–case management of pneumonia and home–based neonatal care. Although HAS and CRHP have been the site of important research, these two projects have had less of a research orientation and more of a service orientation.

The Matlab MCH–FP project differs importantly from the other three in that it functions within the strong institutional framework of an international research center without an obvious single strong small set of long–term leaders. The projects each had two key individuals who led them from the beginning over a long period of time. CRHP is notable compared to the others in its deep commitment to field–based education of thousands of people from around India and beyond who have come to learn about CRHP’s approach to working with communities and CHWs.

Criticisms have commonly been made of smaller “model” CBPHC projects because (i) they are not sustainable and not scalable since they are dependent on charismatic leadership and (ii) they have not had to deal with the management and logistical challenges of operating a program at larger scale, which are an order of magnitude more difficult. From the standpoint of these four projects, it is obvious that they are sustainable because of their long–term operation. None of these projects attempted to go to scale, but in every case certain elements of each project have in fact been scaled up in an indirect sense. The Matlab MCH–FP Project, once proven effective, served as the model of government national scale up of CHW programming. HAS’s approach to CHWs has been adopted by virtually all other NGOs providing community–based services throughout Haiti. The CRHP approach to CHWs has served as a model for national CHW programming in India, both with the Village Health Guides program of the 1980s and the more recently established ASHA program. SEARCH’s demonstration of the effectiveness of community–based management of pneumonia and of home–based neonatal care interventions stimulated further independent confirmatory research. Now, this approach has become the global standard of care around the world in resource–constrained settings, and its home–based neonatal care intervention has guided national replication and scale up by the government of India.

## CONCLUSIONS

Although there is strong evidence that in highly controlled settings, specific interventions such as hand washing, vitamin A, immunizations and many others can improve child health, there is much less evidence for how health projects in high–mortality, resource–constrained settings can achieve long–lasting impacts on under–five mortality. The common characteristics of the four projects cited here give some important insights in considering this important question.

Most of the evidence regarding the effectiveness of community–based primary health care (CBPHC) in improving maternal, neonatal and child health (MNCH) outcomes comes from assessments of the effect of single interventions implemented in highly controlled and atypical field settings over a relatively short period of time, usually five years or less. The four projects identified and described here are the only four in our database of 698 assessments that have evidence of long–term impact of 10 years of more on mortality. The projects described here provide a comprehensive array of child health and maternal and reproductive health services, including family planning. They all provide general curative care, including hospital services, and they all facilitate referral and counter–referral services. Each project uses CHWs and provides strong training and support for them. Each also provides essential services for children in the home, has developed and sustained a high level of community engagement, and has earned the trust of the people it serves.

Each of these projects has used recurrently a cycle of consultation/planning, implementation, reflection/evaluation on a regular basis to adjust their projects to serve the needs of their local population. Over a long time, this process led to project characteristics that have been maintained. The similarity of these four projects’ characteristics attests to the strength of this combination of project characteristics in serving its population’s health needs.

Building strong and more comprehensive health systems in high–mortality, resource–constrained settings along the lines of the projects described here has the potential for not only long–term improvements in MNHC but also long–term improvements in control of HIV/AIDS, malaria and tuberculosis and even many emerging chronic conditions such as hypertension and diabetes. Learning to build and maintain these systems in a way so that they are affordable with local resources is one of our great challenges.
